# Targets and Mechanisms of Photodynamic Therapy in Lung Cancer Cells: A Brief Overview

**DOI:** 10.3390/cancers3011014

**Published:** 2011-03-03

**Authors:** Angela Chiaviello, Ilaria Postiglione, Giuseppe Palumbo

**Affiliations:** Department of Biologia e Patologia Cellulare e Molecolare “L. Califano” - Università Federico II, Via S. Pansini, 5 80131 Naples, Italy; E-Mails: angelachiaviello@libero.it (A.C.); ilariapostiglione@interfree.it (I.P.)

**Keywords:** photodynamic therapy, photosensitizers, lung cancer cells

## Abstract

Lung cancer remains one of the most common cancer-related causes of death. This type of cancer typically develops over a period of many years, and if detected at an early enough stage can be eliminated by a variety of treatments including photodynamic therapy (PDT). A critical discussion on the clinical applications of PDT in lung cancer is well outside the scope of the present report, which, in turn focuses on mechanistic and other aspects of the photodynamic action at a molecular and cellular level. The knowledge of these issues at pre-clinical levels is necessary to develop, check and adopt appropriate clinical protocols in the future. This report, besides providing general information, includes a brief overview of present experimental PDT and provides some non-exhaustive information on current strategies aimed at further improving the efficacy, especially in regard to lung cancer cells.

## 1. Photodynamic Therapy in Cancer

### 1.1. General Concepts

Photodynamic therapy (PDT) is a minimally invasive approach used in oncology that is based on the administration (systemic or topical) of a photosensitizer (PS) agent followed by light activation. The ensuing rapid and intense oxidative burst, results in cytotoxic toxic effects that, in ideal conditions, kill tumor cells sparing the healthy ones.

Since its initial application, PDT continues to find increasing application to treat or relieve the symptoms of selected cases of certain types of malignancies including esophageal, head and neck, breast, prostate, bladder, skin, and lung tumors. Numerous clinical trials are under way to evaluate its use for cancers of the brain, prostate, cervix, and peritoneal cavity (pancreas, intestines, stomach, and liver).

In more recent years, researchers have continued to explore new strategies to improve the effectiveness of PDT, whose adverse effects are essentially limited to prolonged residual photosensitivity. Initially the major research focus was on the development of more powerful photosensitizers. Researchers have also investigated how to improve the light sources and the characteristics of the activating light that can penetrate tissue and treat deep or large tumors. A more recent field of investigations considers the possibility of merging PDT with other conventional therapies as radio, immuno- or chemo-therapy. The rationale of this combined approach is based on the possibility of reducing the dose of the most toxic component (drug, X radiation) preserving the overall therapeutic efficacy. Selective photosensitizer delivery is another current field of investigation.

The word “phototherapy” was introduced about one century ago to indicate the treatment of cutaneous tuberculosis with ultraviolet light [1,2]. Just a few years later researchers observed that a combination of light and certain chemicals could be exploited to induce tissue-damage. In this regard, von Tappeiner and Jesionek [3] used eosin and white light to kill skin tumor cells in patients. However, the current concept of photodynamic therapy and subsequent clinical use has began more recently, less than 50 years ago, thanks to the pioneering work of Thomas Dougherty. In this paper the authors showed that the topical administration of a photosensitizable molecule, namely hematoporphyrin IX, and red light, successfully eradicated mammary and bladder tumor in mice [4]. Following this initial success in animal models, PDT was introduced in human therapy. Since then several trials, almost everywhere in the world, have been conducted to evaluate the use of PDT for cancers of the brain, skin, prostate, cervix, intestines, stomach, and liver, lung and others. Unfortunately, in many cases, success has been considered only partial. Today, except a few exceptions, PDT in clinics is used to eradicate pre-malignant, selected early-stage cancers and to reduce the tumor size in end-stage cancers.

The question is: can PDT be further improved? Could it find increased efficiency when used in combination with conventional therapy? May it strike exclusively and specifically the cancer cells, sparing healthy ones? To answer these questions, a lot of work has to be done with regard to several aspects of PDT including having a better knowledge of the nature of the tumor cell, the nature and properties of the photosensitizer, characterization of specific sub-cellular targets of PDT and understanding the effective cytotoxic mechanism.

### 1.2. Photosensitizers

Among the several photosensitizers (PS) available, very few have been selected for clinical trials owing to many important factors. They include selectivity in terms of target cells *versus* healthy cells, suitable extinction coefficients and accumulation rates in target tissues, stable composition and a chemical nature that may facilitate the entrance in the cell avoiding precipitation in aqueous environments [5]. The recent introduction of nanocarriers has partially modified this view, in that the properties of the PS may be not so important and that specificity toward target tissues may be improved using specific drug delivery strategies, whose detailed discussion is out of the scope of the present review [5].

Photosensitizers are generally classified as porphyrins or non-porphyrins. Porphyrin-derived PS, in turn are classified as first, second or third generation PS.

First-generation PS are hematoporphyrin, its derivative HpD, and the purified, commercially available and yet largely employed Photofrin. This molecule originally approved for use in humans in 1993 in Canada, is now the PS most commonly used in Europe for the treatment of advanced stage lung cancer; in Japan and Europe for early-stage oesophageal, gastric and cervical cancer; and in the United States for advanced esophageal cancer [6,7].

Beside the absence of intrinsic toxicity, other advantages offered by Photofrin include the possibility of using small drug doses, the good clearance from normal tissue and possibility of repeated administrations without serious consequences (but prolonged photosensitivity) for the neoplastic patient [8,9].

The “second generation” PS include benzoporphyrin derivative, chlorins, phthalocyanines and texaphrins as well as naturally occurring compounds, such as hypericin, and substances that promote the production of the endogenous protoporphyrin IX (PpIX) as 5-aminolevulinic acid (5-ALA) and some related esters [10].

Delta (or 5)-aminolevulinic acid (5-ALA) is a stable molecule [11] that behaves as a pro-drug, since it is metabolically converted to the photo-sensitizable protoporphyrin IX. Both 5-ALA and PpIX are naturally occurring intermediates in heme biosynthesis. Normally, heme inhibits the endogenous formation of excess 5-ALA by a negative feedback control mechanism, thereby avoiding natural PpIX photosensitization [12]. However, the presence of exogenous 5-ALA bypasses this regulatory mechanism and results in the intracellular accumulation of PpIX. Following application (that can be systemic or topical, as required), high concentrations of the potent endogenous PS protoporphyrin IX (PpIX) are generated in neoplastic cells that become sensitive to light. In addition to good tumor selectivity, 5-ALA-induced PpIX is characterized by limited systemic toxicity and low skin photosensitization [13]. It is interesting to underline that several Authors have reported that PpIX accumulation is made easy in tumors because the particular porphyrin metabolism in malignant tissues that are characterized by high cellular turnover [14-18].

Besides the peak corresponding to the Soret band at about 405 nm, PpIX has additional absorption peaks namely at 510, 545, 580 and 630 nm (Q-bands). The last wavelength has found application in clinics because red light penetrates into the skin deeper [19].

Hypericin is a phenanthro-perylene-quinone, naturally occurring in plants of the genus Hypericum, especially Hypericum perforatum. Hypericin salts produce wine-red solutions in organic solvents with absorbance maxima around 595 nm [20].

The photosensitizing properties of hypericin were first recognized in animals observing cutaneous photosensitivity following the ingestion of very large quantities of Hypericum plants and exposure to sunlight [21].

Although hypericin is an interesting alternative to chemically synthesized photosensitizers, it absorbs in a spectral region in which the light penetration is limited. Hypericin, in fact, has an action spectrum that peaks around 595 nm, and does not absorb light above 630 nm. Attempts to shift the absorption spectrum of hypericin by chemical modification achieved only partial success so its potential in clinical PDT mainly lies in the treatment of superficial lesions [22,23].

Ongoing clinical work is addressing the potential of hypericin as the PS of choice in bladder cancer PDT. This is because of its attested, specific accumulation in urothelial carcinoma lesions and its proven safety and efficacy as a diagnostic tool when administered intravesically [24,25]. Reports on the localization of hypericin following cellular uptake indicate a general association with lipid membranes including the endoplasmic reticulum the Golgi apparatus [26] and lysosomes [27]. Currently hypericin alone or in combination is the object of some interesting *in vitro* [28] and *in vivo* [29,30] investigations which have important implications for recurrent breast cancer therapy.

An extremely potent second generation PS approved in Europe for the palliative treatment of neck and head cancers is the meso-tetra-hydroxyphenyl-chlorine (named Foscan or Temoporfin). This molecule, which has been shown to have a short plasma half-life in humans, is hydrophobic in nature, is strongly photoactivable at 652 nm with very high singlet oxygen yield while appearing to preferentially accumulate in tumor cells [2,31-33]. In addition, beside a direct damage to tumour cells, the curative effect of Foscan is also attributed to its pharmacokinetic behavior that causes intense and sustained vascular damage [34].

Another PS that deserves particular mention is Talaporfin sodium (TS) ((+)-tetrasodium (2S,S)-18-carboxylato-20-[N-(S)-1,2-dicarboxylatoethyl]-carbamoylmethyl-13-ethyl-3,7,1,17-tetramethyl-8-vinylchlorin-2-propanoate) a second-generation PS with a core chlorin structure containing a highly aromatic system. The very good water solubility and the shorter half-life make TS an attractive drug [35]. In preclinical experiments, activation of Talaporfin sodium with laser light at 664 nm generated singlet oxygen in a drug dose-dependent fashion. The depth of treatment is dependent on the ability of light to penetrate the target tissue with enough photons to activate the drug. Singlet oxygen causes significant alteration of macromolecules via oxidation of biological substrates such as DNA, membrane lipids, cholesterol and solvated molecules [36]. Preclinical studies have demonstrated that TS activation induces also systemic, tumor-specific immuno-modulation mediated by CD8+ T cells which involves up-regulation of both cytolytic and memory cells [37] and microvessels closure that may help in overcoming tumor resistance [38,39].

An incomplete list of photosensitizers used in clinics, clinical trials or at preclinical stages is indicated in Table 1.

## 2. Curative PDT

### 2.1. Fundamentals

PDT requires the administration and the selective accumulation of a photosensitizer in cancer tissue followed by exposure to suitable light. Following the absorption of photons, the energy-enriched photosensitizer renders back the excess energy and returns to its ground state. Because of this process, the excess energy can be released to surrounding substrates.

Importantly, a fraction of the excited singlet state molecules is transformed via *intersystem crossing* into the relatively long-lived (micro-to milliseconds) excited triplet state, which can either form free radicals or radical ions by hydrogen atom extraction or electron transfer to biological substrates (such as membrane lipids), solvent molecules or oxygen.

These radicals can interact with ground-state molecular oxygen to produce reactive oxygen species (ROS) as superoxide anion radicals, hydrogen peroxides, hydroxyl radicals and others. Such a reaction is generally indicated as Type I. Alternatively, from the excited triplet state, the compound can transfer directly its energy to ground-state molecular oxygen to form highly reactive singlet oxygen. Such a reaction is generally indicated as Type II. Indeed, both these reactions occur simultaneously. The ratio between the two reactions, however, depends on the specific nature of the PS and substrate [40]. Certainly the ^1^O_2_ plays an important role in all molecular processes initiated by photo-activation [41].

It is clear that Photodynamic action only strikes cells that are proximal to the site of the ROS production (as determined by the PS instant localization) whereas surrounding tissues are largely spared. This is due to the fact that in biological systems the half-life of ^1^O_2_ is very short (≤ 0.04 μs), and, therefore, its radius of action is less than 0.02 μm. The ROS that are generated during PDT have been shown to destroy target tissues by multifactorial mechanisms. In fact, the therapeutic response of PDT depends on a complex combination of variables strictly related to the experimental system considered as a whole. In this regard, drug dose, drug-light interval, tissue, tissue oxygenation, light dose and light intensity (the last two are more accurately referred to as fluence and fluence rate, respectively), have to be considered.

PDT can eradicate malignant cells by direct tumor cell kill, vascular shutdown and immunologic effects. Indeed, ROS-mediated photo-damage induces tumor destruction through three principal mechanisms, two of which are direct consequences of ROS production. The first, and more relevant, is the cancer cell killing due, as already stated, to intracellular explosive generation of high reactive ROS, while the second is represented by the possible destruction of the tumor-associated vasculature. A third therapeutic effect has been assigned to a possible activation of an immune response. The relative importance of each of these mechanisms in contributing to the long-term tumor control is not easy to establish. Certainly, the final therapeutic outcome depends on several factors that include the particular type of the tumor and vasculature, the nature of the PS, its concentration and localization, the time interval between the administration of the drug and the light exposure, the light fluence (the total energy of exposed light across a sectional area of irradiated spot), the fluence rate (the radiant energy incident per second across a sectional area of irradiated spot), the local oxygen availability and many more factors.

### 2.2. Direct Tumor-cell Killing

The mechanisms by which the photodynamic action may cause direct cell killing are various and span from necrosis, to apoptosis or autophagy [42]. A key factor in determining ultimate cell fate, *i.e.*, the balance between apoptosis and necrosis, is the intracellular localization of PS that strictly depends on its chemical nature and the light fluence [43,44]. The short-life and spatially limited diffusion of singlet oxygen imply that primary molecular targets of the photodynamic process must be located within a few nanometres from the intracellular site of photosensitizer localization. Several observations have indicated that compounds that localize within mitochondria or ER promote apoptosis, while activation of PS targeting either the plasma membrane or lysosomes can either delay or even block the apoptotic program prompting the cells to necrosis [44]. *In vitro*, the distribution of a PS within a cell depends also on the concentration of the dye in the culture medium and the extent of the incubation time. For example, the widely used PS photofrin has been shown to concentrate into plasma membranes or cytoplasm upon brief incubation, and in the Golgi complex or ER upon prolonged incubation [45]. Other studies have reported even on the re-localization of certain PS after irradiation suggesting that, besides the primary sites, photodamage can be directed to other subcellular locations [46,47].

Obviously even light fluence determines cellular response to PDT: in general, necrosis appears to be the predominant mode of cell death when cells are strongly photosensitized, while apoptosis becomes the principal cell death modality when photosensitization is not extensive (light fluence and/or PS concentration) [48]. In the latter case, however, apoptosis has to compete with autophagy (shortly outlined below). In fact sublethal PDT may trigger, through this mechanism, the removal of damaged organelles and promote cell survival [49]. Although, autophagy has been described *in vitro*, it is however conceivable that it may also occur *in vivo*.

### 2.3. Vascular Damage

The viability of cancer cells depends on the amount of nutrients supplied by the blood vessels. In turn, formation and maintenance of blood vessels depend on growth factors produced by tumor or host cells. An approach currently used to treat cancer is the targeting of tumor vasculature directly (*i.e.*, damaging the endothelial cells) or indirectly (*i.e.*, targeting factors that stimulates cell division and the growth of new blood vessels) [50]. PDT, indeed, has an effect on the tumor vasculature, in that photosensitization through the ROS production may cause the shutdown of vessels consequently depriving the tumor of nutrients. In particular, MV6401 (pyropheophorbide derivative)—a second generation photosensitizer—has been proved to elicit a biphasic vascular response in experimental animals following photodynamic treatment. The most rapid response observed was vasoconstriction followed by a late formation of a thrombus [51] and necrosis. These vascular effects were associated with a delay in tumor growth. Similar effects have been described earlier also with photofrin in rats bearing experimental chondrosarcoma [52]. Nuclear magnetic resonance imaging studies using *in situ* fluorine have demonstrated that damage to the tumor vasculature precedes tumor necrosis [53].

Interesting enough, vascular endothelial growth factor (VEGF) and cycloxygenase (COX-2)—both potent angiogenic factors—were up-regulated during PDT [54]. As a consequence, the concomitant use of specific inhibitors of these factors may positively influence the outcome of PDT, when used with the aim of targeting vasculature.

### 2.4. Immune Response

In contrast to most cancer treatments that are in general immunosuppressive, in certain conditions PDT may exert a pro-inflammatory action. For example it has been demonstrated that PDT causes invasion and leukocyte infiltration of the tumor, events that are typical of acute inflammation and immunity. In addition, PDT appears to increase the presentation of tumor-derived antigen to T cells [55]. Simultaneously, PDT stimulates the recruitment of host leukocytes, lymphocytes, neutrophils and macrophages into tumor tissue, by up-regulating the inflammatory cytokines interleukin IL-6 and IL-1 [56,57].

The expression of heat shock proteins (HSPs) is enhanced too. For example, it has been shown that following sub-lethal photodynamic treatment of MCF-7 cells, the HSP70 mRNA level was significantly increased [58]. It has been reported that HSP70, released from necrotic tumor cells, inhibits tumor apoptotic cell death and promote the formation of stable complexes with cytoplasm tumor antigens. These antigens can then be either expressed at the cell surface or run away from dying necrotic cells, interact with antigen-presenting cells favoring an antitumor response [55,59].

Another interesting observation on long term effects of photofrin/PDT was concerned with the major tumor recurrence in immuno-compromised Balb/c mice as compared to the normal counterpart. Interesting enough, this effect was reversed by bone-marrow transplants from immuno-competent donors [57]. Another interesting study concerning the immuno-stimulatory effect of PDT reports that extracts from Photofrin/PDT treated cells were successfully exploited as vaccine in mice against the development of further tumors [60]. In conclusion the immune response that occurs later after a photodynamic treatment may help in improving the overall therapeutic efficacy by eliminating the survived cells.

## 3. Subcellular Targets of PDT

The main damages induced by PDT occur where the photoactivable agents localize within the cell [61]. Sensitizers can accumulate almost everywhere within the cell; there they cause specific detrimental effects. Nevertheless, mitochondria and endoplasmic reticulum represent the preferential targets [6].

### 3.1. Mitochondria

Involvement of mitochondria in PDT has been object of several studies. The earliest events observed after photoactivation are the cytochrome C release from mitochondria [62] and the subsequent rapid induction of apoptosis [63,64]. According to some authors [6] the variations of intracellular Ca^2+^ is responsible of the increase in mitochondrial membranes permeabilization. The discharged cytochrome C participates in the formation of the apoptosome complex that ultimately activates caspase 3.

Other studies demonstrated that ROS generated by photoactivation, can also activate caspases 8 and 3 through the induction of the FAS/TNF receptor multimerization [65].

Other research groups have focused their attention on the function of Bcl-2 a protein, which is principally present in the outer membrane of the mitochondria, but also in endoplasmic reticulum and the nuclear envelope.

It has been reported that PDT causes *in vivo* and *in vitro* a down-regulation of Bcl-2 antiapoptotic activity. To explain this behavior it has been proposed that Bcl-2 may undergo a direct oxidative damage (yielding to a conformational change) from PDT-generated ROS [65].

On the other hand, it was shown that the overexpression of Bcl-2 prevents (by inhibiting the cleavage of pro-caspase 3 and 9) PDT-mediated-DNA fragmentation and apoptosis in Chinese hamster ovary cells [66]. Coherently, the transfection of antisense Bcl-2 retrovirus vector increased the sensitivity of a human gastric adenocarcinoma cell line to photodynamic therapy [67].

On the whole, these studies may indicate that PDT may induce specific effects in which Bcl-2 is either a direct target of radicals, or a downstream effector of signal transduction. In any case the function of Bcl-2 still remains somewhat elusive.

### 3.2. Endoplasmic Reticulum

In eukaryotes, the role of endoplasmic reticulum (ER) is linked to two specific functions: a) Ca^2+^ storage and signaling and b) folding, remodeling and sorting of newly synthesized proteins. Disturbances in any of these functions can lead to ER stress. It is now known that ER stress may determine changes in protein folding and subsequent activation of the unfolded protein response (UPR).

The UPR is a primarily pro-survival response activated to reduce the accumulation and aggregation of unfolded or misfolded proteins and to restore normal ER functioning prevalently by the induction of molecular chaperones [68]. However, if ER stress persists, the UPR can activate a cell death program, which often requires the activation of caspases cascade.

Recent data indicate that following PDT, different heat shock proteins as well as the ER chaperones, GRP78/Bip, calreticulin, calnexin are induced in a time dependent manner [69]. It has been also demonstrated photoactivation may cause the induction of c/EBP homologous protein (CHOP), activation of the ER stress-mediated caspase-12 and apoptosis [70]. A more recent genome-wide analysis in hypericin-PDT exposed bladder cancer cells revealed that molecular sensors and effectors of the UPR are induced coordinately [71].

These studies indicated that perturbations in the ER caused by accumulation of photo-oxidated proteins can sustain the activation of UPR pathway.

The body of data available so far, however, does not allow a detailed comprehension of the functional impact of the UPR on the modulation and efficiency of PDT-induced cell death both *in vitro* and *in vivo* systems. Therefore, a more accurate and critical evaluation is required.

### 3.3. Cytoplasm

Another target of PDT is the cell cytoskeleton [72,73]. For example it has been demonstrated that 5-ALA/PDT led to the disruption of the cytoskeleton structure. The uniform architecture of actin filaments was destroyed, due to possible alterations of several cytoskeleton organizing proteins. The perturbation of actin filaments induced by 5-ALA/PDT involves the suppression of the adapter protein PDZ-LIM and Cofilin dephosphorylation, both enabling actin depolymerization. An additional contribution in abolishing actin organization is finally determined by Septin2 suppression, whose filaments are essential in stabilization of actin stress fibers [74].

PDT also decreases cell attachment to a substratum as observed in various systems. For example photosensitizations with benzoporphyrin derivative-monoacid ring (BPD-MA) and 5-ALA were reported to inhibit cell attachment to components of the extracellular matrix as fibronectin, vitronectin, and collagen P [75]. Interesting enough the cell adhesion process was impaired not only by massive photosensitization (high light fluence rate), but also by moderate exposures that spare a large number of cells. The observed delay in the attachment of surviving cells was associated to alterations in the cell adhesion machinery (*i.e.*, integrins, other scaffold proteins, intracellular signaling system, *etc.*), caused directly by PDT [76].

Other observations on cells survived to PDT indicated also that some important morphological changes were occurring with time [6]. Such changes, often consisting of extensive surface blebbing, loss of microvilli and in the emission of filopodia, were clearly related to changes in the cytoskeleton organization. In this regard tubulin network around the nucleus was disarranged while bundles of actin microfilaments became progressively thicker facilitating cell detachment [77].

It is possible that the effect of PDT on perinuclear structures (including mitochondria) may influence the remote adhesion processes at the cell surface [78]. This may occur because changes in the cell shape, reorganization of the cytoskeleton and even signal transduction, are part of cellular responses that originate in other cellular compartments [79]. In this regard, the links between the intracellular domain of integrins, the cytoskeleton and signaling molecules such as focal adhesion kinase, src, kinase and others are unquestionably crucial [80].

It also reasonable to hypothesize, that the observed reorganization of cellular cytoskeleton is a consequence of the beginning of a PDT-mediated apoptotic program [73].

The decrease in the adhesiveness of cancer cells after PDT is an interesting fact since it has been used to explain the reduction of the metastatic potential of surviving tumor cells [81,82]. Indeed, this inhibition has been reported repeatedly [83,84] and may represent an appealing advantage of PDT over other therapeutic approaches [85].

### 3.4. Nucleus

The involvement of the nucleus in PDT is unclear. For example, it has been reported that the observed DNA fragmentation was not a direct effect of photosensitizer accumulation within it, but rather an indirect effect due to the action of ROS generated on the nuclear membrane [86]. Similarly, other authors attributed the observed enlargement of the nuclear membrane to an influx of water caused by PDT-mediated Ca^2+^ transport impairment [87]. Recently it has been reported that the photoactivation of a specific photosensitizer, namely 5,10,15,20-Tetrakis (*N*-methyl-4-pyridyl)-21H,23H-porphyrin, caused a direct DNA damage [88]. These authors reported that exposure of cells to light produced measurable amounts of 8-oxo-Guanine, a typical product of DNA oxidative damage [89].

It is important to emphasize that the nucleus is a very sensitive target for reactive oxygen species [90,91]. In this regard, a targeted delivery of photosensitizers to the nucleus should be seen as a powerful way to potentiate the effectiveness of PDT as tumor-cell killing strategy [91].

### 3.5. Plasma Membrane

Binding of porphyrins to the plasma membrane was observed for the first time in an *in vitro* system by Moan *et al.* using fluorescence microscopy [92]. Among the first PDT-mediated effects on plasma membrane reported in different cell lines were the K^+^ leakage and the inhibition of trans-membrane transport systems [93]. Other effects included the cross-linking of membrane associated polypeptides, the inactivation of specific membrane-bound enzymes, inactivation of receptors and impairment of ion channels [94].

More recently Hsieh *et al.* found that the distribution of photofrin was dynamic in human epidermoid carcinoma A431 cells and was dependent on the incubation time. In particular, it was observed that the damage of plasma membranes induced necrosis-like death. In contrast, when the plasma membranes were undamaged cells were characterized by cytoplasmic vacuoles formation and cell shrinkage/fragmentation. It has been also demonstrated that the damage of plasma membranes was related to the intracellular ROS formation triggered by PDT [45]. Among the several downstream signaling events following the generation of ROS in the vicinity of the plasma membranes, the activation of JNK and caspase 3, cleavage of PARP have been repeatedly reported [95-97]. Procedures that selectively lock up photosensitizer to the plasma membranes of transformed cells might be more successful in PDT treatment of tumors that infiltrated or metastasized [45].

## 4. PDT in Lung Cancer Cells

When high doses of light directly hit a cancer cell, sufficient oxygen radicals are generated to kill cells rather instantaneously. However not all cells in a tumor are exposed directly to light. Inner cell layers, in fact, are reached by progressively lower light doses. In this instance the commitment events as well as the modality of cell death induced by PDT are more complex.

Below an overview of the principal cellular and molecular effects that have been observed is reported.

### 4.1. Instant and Delayed Lethal Effects

#### 4.1.1. Necrosis

Necrosis is the major cell death modality induced by PDT with compounds localized to the plasma membrane [6,44]. In lung cancer cells the “prompt” cell death upon photofrin or zinc(II) phthalocyanine photosensitization is likely due to a fast loss of plasma membrane integrity, incapability to maintain ion fluxes across the plasma membrane and rapid depletion of intracellular ATP [45]. Although the biochemical pathway inducing necrosis following PDT has not been clearly recognized yet, some factors, such as increased intracellular Ca^2+^ and/or the specific generated ROS, may be crucial in promoting necrotic cell death. In mice transplanted with TC-1 lung cancer cells the mode of cell death induced by PDT was almost entirely confined to necrosis and essentially site-specific with respect to the distribution of a hematoporphyrin derivative (Photogem) used as photosensitizer [98]. In contrast, apoptosis was the preferred mode of photo-killing in lung cancer cells when PpIX was associated to the mitochondria [99].

#### 4.1.2. Apoptosis

Apoptotic cell death is incontestably the best-studied form of programmed cell death, having implications in several physio-pathological conditions and in therapeutic response [100].

Apoptosis is an energy-consuming procedure that requires the coordinated activation of proteases and nucleases leading to degradation of intracellular structures and DNA break-ups without inducing inflammation [101]. The apoptotic process entails the activation of a family of cysteine dependent aspartate-specific proteases (caspase) [102] that, upon specific stimuli, are activated by multimerization (initiator caspases) or limited proteolysis (effector caspases) [103]. The latter are considered responsible for most of the apoptosis peculiar morphological and biochemical changes. The process of apoptosis is controlled by several signals, which may originate either within the cell (intrinsic pathway) or outside it (extrinsic pathway) [104].

##### 4.1.2.1. Intrinsic Pathway

Mitochondrial permeabilization plays a pivotal role in the intrinsic pathway of apoptosis. Since several photosensitizers demonstrate significant affinity for mitochondria, the permeabilization of their membranes upon photoactivation is not an unexpected event [12,61].

Studies *in vitro* with human invasive lung carcinoma cell lines demonstrated that 5-ALA/PDT induced [105] a reduction of mitochondrial function through a manifest modification of mitochondrial membrane potential that induces a reduction of cellular invasiveness. In agreement with findings obtained on lung cancer cells, other studies, on different transformed cells reported a direct effect of PDT on the rapid reduction of mitochondrial membrane potential and a concomitant activation of caspases 8 and 3 [106].

A second mechanism for mitochondria membrane permeabilization entails the formation of proteic channels in the outer mitochondrial membrane through a process involving oligomerization of pro-apoptotic Bcl-2 family members [107]. This family encompasses several proteins, all critical for the regulation (positive and negative) of apoptosis [108].

Two proteins of this family have been best studied in PDT. They are Bax, a protein essential for the release of cytochrome C from mitochondria and key player in inducing apoptosis and the antiapoptotic protein Bcl-2 [44].

We have specifically analyzed the expressions of pro/anti-apoptotic proteins in human H1299 and A549 lung adenocarcinoma cells upon PDT.

In these systems we confirmed, in agreement with findings of other authors [109], that photoactivation induced a reduction in Bcl-2 expression. However, at variance with previous findings, we noticed that this down-regulation was followed by a visible increase [110]. We attributed this biphasic behavior to a transient proteasome block. Since the initial proteasome impairment did not prevent the synthesis of new Bcl-2 molecules, they continued to accumulate until the newly synthesized Bcl-2 molecules in excess, exerted a negative feed-back action. Within two or three additional hours the proteasome appeared to recover as indicated by the abrupt, reported fading of Bcl-2 expression [110].

The function of the propapoptotic Bax protein in photoactivated lung cells has been studied as well. For instance, Wu S. *et al.* demonstrated that Photofrin-PDT induced in lung adenocarcinoma cells the up-regulation of Bax resulting from an initial mitochondrial depolarization [99]. It has been also demonstrated that in Lewis lung carcinoma cells that mono-L-aspartyl chlorin e6/PDT induced cytotoxicity because the balance between anti(Bcl-2) and pro apoptotic (Bax) proteins was shifted in favor of the second [111].

In certain PDT scenarios it has been suggested that Bid, another member of the Bcl-2 family, may be involved. In details, it has been reported that Bid is strongly implicated in Bax and Bak activation leading to cytochrome C release and apoptosis [112]. Another similar observation in human lung adenocarcinoma cells, that concerns the involvement of Bid as pro-apoptotic factor, has been reported by Wan *et al.* These authors, in fact, described that the silencing of Bid remarkably inhibited NP6/PDT-mediated apoptotic cell death [113].

##### 4.1.2.2. Extrinsic Pathway

Several reports have indicated the evocation of the Fas and Fas-ligand system upon PDT both *in vitro* and *in vivo* systems [10,114], suggesting that the extrinsic pathway of caspase activation contributes to the PDT-mediated apoptotic response.

A combination of PDT with Photofrin and TNF-α resulted in an increased tumor killing in mice compared to individual therapies [115]. Similarly an additive effect on murine YAC-1 lymphoma cells has been described in a treatment combining TNF-α with Photosan 3/PDT [116].

More recently it has been reported [117] that silicon Phtalocyanine c4/PDT induced the multimerization of Fas protein, the protein expression of Fas ligand, FADD, an adapter molecule for Fas, and the binding of FADD with Fas. These data delineated an ultimate involvement of the Fas pathway as an important contributor to photodynamic-therapy-mediated apoptosis of cancer cells. Such overexpression was also observed *in vivo* in tumor-bearing mice [114] following Photofrin/PDT.

Fas-ligand expression plays a role in activating apoptosis also in human lung cancer cells undergoing photoactivation. In this regard, Olivo *et al.* demonstrated that Fas-ligand was involved in apoptosis signaling mechanisms elicited by Calphostin/PDT [106].

#### 4.1.3. Autophagy

Authophagy (or macroautophagy) is a physiologic process by which cytosol and entire organelles coalesce forming a vacuole termed autophagosome. Within the cell these vacuoles rapidly merge with lysosomes and undergo degradation. Although autophagy has been at first described as a survival response to nutrient deficiency, as the removal of damaged organelles by this mechanism may help promoting cell survival [49], several recent studies suggest that autophagy can also serve as a cell death pathway [118].

Up to now, no data concerning autophagy are available on human lung cancer cells. Recently, however, this physiological process has been described in PDT-treated leukaemia cells [119].

#### 4.1.4. Senescence

Inhibition of apoptosis has been reported to have little or no effect on clonogenic survival after treatment with anti-neoplastic drugs or radiation in several tumor cell lines [120]. Apparently such change is characterized by an increase in the fractions of cells that undergo permanent growth arrest with peculiar phenotypic features of senescence [121]. This condition is, in general, characterized by significant modification of cell morphology (enlarged), increase of specific enzymatic activity (Senescence Associated acid beta-galactosidase) and over-expression of cell cycle inhibitors (p21 and/or p16) [122]. Up to now, we are not aware of papers describing the appearance of these features in cancer cells upon photodynamic treatment. However, unpublished data from our laboratory demonstrate that a premature senescence program may be activated in A549 and H1299 human lung adenocarcinoma cells in particular photoactivation conditions [123].

### 4.2. Cellular and Molecular Effects in Cells Surviving PDT

#### 4.2.1. Cell Cycle

The ultimate effects of a photodynamic treatment on cancer cell lines depend on several factors. Efficient cell death (necrosis or apoptosis) is observed when light, oxygen and the photosensitizer are not limiting (“high dose PDT”). When one of these components is defect (“low dose PDT”), cells may face different fates including apoptosis, autophagy and senescence through activation of specific molecular pathways. Most of them require an initial cell cycle arrest. Up to now only limited data report cell cycle arrest after PDT in lung cancer cells and most of them have been obtained in our laboratory. In this regard, studying non-small cell lung cancer cell line H1299, we observed that Photofrin/PDT targeted G_0_/G_1_ phase inducing simultaneously a significant reduction in Bcl-2 expression [124]. This finding coincides with previous observations reporting that the light activation of specific photosensitizers involve Bcl-2 degradation [109]. In A549 and H1299 lung adenocarcinoma cells, furthermore, we have demonstrated that PDT, causing an “early proteasome malfunctioning” induced a time limited G_2_/M phase arrest [110].

In general, however, literature data about cell cycle after PDT treatment in other cancer cell lines were contrasting. Vantieghem *et al.* demonstrated that hypericyn/PDT in HeLa cells caused a CDK1-mediated G_2_/M arrest and Bcl-2 phosphorylation in a dose and time-dependent manner delaying the onset of apoptosis [48].

In contrast G_0_/G_1_ arrest was observed upon photosensitization in other systems. For example, we observed accumulation of human breast cell cancer cells (MCF7) in this phase upon photosensitization with indocyanine green and infrared laser. This arrest was accompanied with the up-regulation of p21 and p53 expression levels and transient destruction of Bcl-2 [125]. Similar observations were reported by Ahmad *et al.* studying silicon phthalocyanine Pc4/PDT photoactivation in A431 human epidermoid carcinoma cells. In this case the observed G_0_/G_1_ cell cycle arrest was associated with inhibition of the expression and activity of the two CDK2 and CDK6 cyclin kinases and of their regulatory partners cyclin E and cyclin D1 [126]. The role of different cyclin upon PDT treatment has been, indeed, more closely investigated. It is known that the D/CDK4/6, A/CDK2 and E/CDK2 complexes are all involved in the phosphorylation of Rb during the cell cycle. The observed decreases in CDKs expressions in photosensitized cells may well explain the reduction in Rb phosphorylation. Such a reduction caused by PDT may ultimately be responsible for the induction of WAF1/CIP1/p21 and the consequent cycle arrest [127]. This has been, in addition, directly observed by photoactivating silicon phthalocyanine Pc4 *in vivo* in OVCAR-3 tumor xenografts (athymic nude) and *in vitro* (human ovarian carcinoma cells) [128].

The hypo-phosphorylated status of Rb, furthermore involved the inhibition of E2F family of transcription factors, with important consequences on cell cycle. In this regard, the photodynamic response of human epidermoid carcinoma A431 cells (lung) sensitized with silicon phthalocyanine Pc4, was accompanied by a down-regulation of all five members of the E2F transcription factor family [129].

#### 4.2.2. MAPK and Akt

Mitogen-activated protein (MAP) kinases are serine/threonine-specific protein kinases that act in response to extracellular stimuli (*i.e.*, mitogens, osmotic and oxidative stress, heat, cytokines) and master cell activity including gene expression, mitosis, differentiation, proliferation, and survival/apoptosis. Three distinct MAPK pathways have been described: the extracellular signal-regulated kinase (ERK) cascade which is usually triggered by mitogens, the c-Jun N-terminal kinase and the p38 MAP kinase cascades that are both activated in response to chemical and environmental stimuli [130,131]. As these kinases are strongly implicated in the biology of cancer cells, their signaling pathways have been chosen as important targets for purposely designed drugs [132,133]. Since MAPKs are sensitive to oxidative stress, it is not surprising that PDT may elicit their activation and function as effectors of cell response [71]. Nevertheless their functions in the regulation of cell death/survival have not been yet fully elucidated, and remain controversial. In fact, these stress kinases have been reported, from time to time, to promote [134] or to protect from apoptosis [135].

Similarly controversial, is the function of Akt kinase. This protein rules other survival pathways that are oxidation sensitive and therefore, also responsive to PDT. As reported in a few studies, it may apparently induce cell survival or apoptosis according to specific conditions determined by variables [136-138]. Besides other vaguely defined conditions, the action of Akt (as that of the stress kinases ERK, p38 and JNK) appear to depend on cell type, photosensitizer used and light doses. As far as lung cancer cells are concerned, no literature data are available. However, preliminary findings from our laboratory regarding the effects of 5-ALA/PDT on two lung adenocarcinoma cell lines indicate that Akt was rapidly upregulated while ERK activation was dependant on the particular cell phenotype [123].

#### 4.2.3. NF-κB

Since its discovery in the late 1980s, a tremendous number of reports have been published concerning the role of the nuclear factor-kappa B (NF-κB) and its implication in a variety of physiological (organ development, cell survival, proliferation and migration) and pathological processes (including cancer).

NF-κB activation has been granted a positive role as it can cause tumor destruction by eliciting the onset of the immune defense. Interesting enough, NF-κB is also involved in the modulation of the expression of antiapoptotic genes which, in apparent contrast with the previous statement, may favor tumor cell survival, especially in response to pharmacological induction of apoptosis [139,140]. Another feature that characterizes NF-κB is its possible involvement in tumor recurrence because it may upregulate the expression of specific factors stimulating proliferation and angiogenesis [141].

As the effects of PDT on NF-κB activation and function in cancer cells are concerned, not surprisingly, contentious conclusions have been gathered [142]. To now this apparent behavioral dichotomy in activity has not yet been fully deciphered; in this regard, however, it has been hypothesized that a particular mechanism of NF-κB activation and successive function depends on the photosensitizer used as well as the specific cell type studied [143].

We are not aware of specific studies on PDT-mediated regulation of NF-κB in lung cancer cells, however, current experimental activity in our laboratory has enlightened an important involvement as pro-survival factor in cell response to 5-ALA/PDT [123].

#### 4.2.4. Proteasome

The synthesis of NF-κB precursor or the degradation of NF-κB suppressor can be regulated by the proteasome [144,145]. Then the proteasome activity strictly influences the NF-κB activity. Proteasome substrates include signaling molecules, tumor suppressors, cell cycle regulators, transcription factors, inhibitory molecules, anti-apoptotic proteins as Bcl-2 and others [146]. When the degradation of these proteins is interrupted, the effect is particularly significant in rapidly dividing cancer cells, which need continuous supply of growth-promoting proteins to sustain the accelerated and uncontrolled proliferation [147]. Inhibition of the proteasome may therefore arrest or retard cancer progression by interfering with the ordered degradation of cell-cycle proteins and other factors, including NF-κB.

PDT and proteasome activity in lung adenocarcinoma cells has been extensively studied in our laboratory [110]. In this study, while it was proven that sublethal Photophrin/PDT reversibly inhibited proteasome activity within a short time following the photosensitization, we demonstrated that inhibition of the proteasome through specific inhibitors (Bortezomib or even Aspirin) could synergistically strengthen the therapeutic effect of photodynamic therapy.

## 5. Current and Expected Approaches

### 5.1. Combination Therapy

Several preclinical studies and some clinical trials suggest that the use of PDT in combination with standard antineoplastic drugs may become a workable anticancer strategy [2,148].

Indeed, this approach may present several potential advantages as, in the most favorable conditions, it may allow the reduction of the dosage of individual drugs and consequently the lessening of important side effects, while the overall efficacy may be preserved or even augmented. This concept may also be applied to a combined therapy that merges PDT with more traditional treatments such as specific drugs. However, combination protocols are far from being established as the final therapeutic outcome, even *in vitro*, it appears to depend on assorted (cellular and/or molecular) factors. Several years ago, it was generically indicated that the combination of Adriamycin and Hematoporphyrin-Derivative/PDT may have potentiated the photodynamic effect in an *in vivo* transplantable mouse tumor assay [149]. More recently, Ma *et al.* [150] demonstrated in a murine model that the combination of Meso-tetra(di-adjacent-sulfonatophenyl)-porphine/PDT with vincristine (a microtubule inhibitor), enhanced antitumor activity. However, it was reported that this favorable event occurred only when PDT was administered to animals within a defined time span. Another combined PDT-based strategy to treat cancer used in preclinical models, proposed a photosensitizable dye conjugated to monoclonal antibodies raised against tumor specific antigens [151]. While some success was reported, some drawbacks are immediately apparent since the use of large molecules (such as monoclonal antibodies) in PDT is complicated by the difficult synthesis of dye-antibody complex and also the potential toxicity which is not easy to predict [152,153].

At a molecular level, we and others observed that doses of drugs used as standard regimen for first-line treatment of advanced non-small-cell lung cancer and Photofrin/PDT were far more effective in killing H1299 cells when used in combination with specific antineoplastic drugs [124]. This paper is the first to report that the additivity in combined therapy takes place often, but synergy occurs only when the two approaches used in combination (*i.e.*, two different drugs or PDT plus a specific drug), exert disjointed activities on cell cycle [124]. This conclusion was also supported by more recent observations on esophageal cells describing PDT in combination with an anticancer drug [154].

### 5.2. Drug Delivery and Nanoparticles

The obvious ways to improve PDT efficacy require the development of new photosensitizers, optimization of protocols and precise dosimetry [155]. However, even for PDT, innovation in drug delivery and precise targeting may be a real breakthrough.

Drug delivery is one of the main challenges in medicine to be overcome today. PDT does not make an exception. In this regard, nanoparticles represent an emerging strategy that show great promise for PDT to carry suitable sensitizers directly to the diseased cell, sparing, if possible, the healthy ones. A major disadvantage of nanoparticles is their susceptibility to be taken up by the macrophages after intravenous administration. However, it has been demonstrated that coating nanoparticles with polyethylene glycol (PEG) enhances their circulation time in the bloodstream, so facilitating the accumulation in tumors [157]. As far as photosensitizers and PDT are concerned, several approaches (namely, liposomes, oil-dispersions, polymeric particles and hydrophilic polymer–PS conjugates, *etc*.) have been proposed, with encouraging results [157]. In particular, it has been recently reported the use of dendrimer phthalocyanine (DPc)-encapsulated non active polymeric micelles as basis for lung cancer-related photodynamic treatment. These nanoparticles have been successfully used, in fact, either *in vitro* or *in vivo* in human lung adenocarcinoma A549 cells and in mice bearing subcutaneous A549 tumors, respectively [158]. In both experimental systems the authors claimed significantly higher PDT efficiency as compared to the traditional modality of drug administration due to the specific photosensitizer localization in cellular mitochondria [158].

A more direct and specific localization of the photosensitizer with increased efficiency and selectivity can be accomplished by active targeting. This approach relies on conjugates that contain a receptor-targeting moiety and photosensitizer that, not only increases the affinity of the binding moiety to the receptor or antigen on the targeted cell surface, but also allows for a lower effective dose of the PDT drug [159].

## 6. Conclusions

Over the last three decades and more, researchers have continued to study new ways to improve the effectiveness of PDT and diffuse its use in human therapy. During this time the enthusiasm has incessantly alternated with coolness. For this we must ask ourselves what does the future hold for PDT? This is difficult to predict but, as this short overview may have indicated, the multidisciplinary nature of this approach allows substantial room for improvement in the near future.

## Figures and Tables

**Scheme 1.**
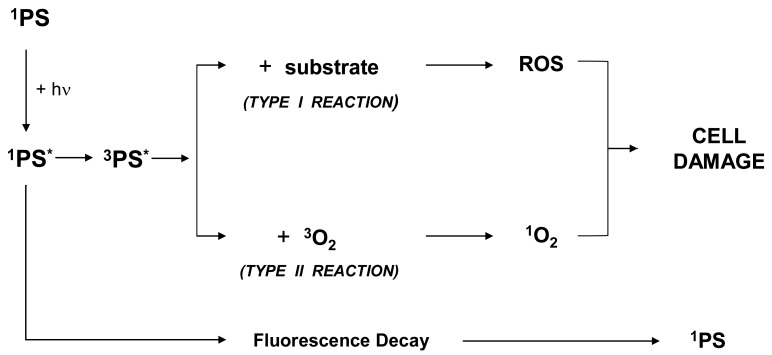
Formation of Reactive Oxygen Species (ROS) according to Photo-Type I and Type II reactions. hν indicates an excited photon; ^1^PS and ^3^PS* denote the photosensitizer in its ground and excited states; all molecular components of a living cell are comprised under the expression “substrate”. ROS (including ^1^O_2_) are the ultimate species responsible for cell damage.

**Table 1.** Photosensitizers commonly used in *in vitro* and *in vivo* studies.

**Photosensitizer**	**Trade name**	**Reported applications**	∼ λ (nm)
Porfimer Na	Photofrin	Cervix, advanced and early lung, bladder, superficial gastric, brain, esophageal cancers.	630
m-THPC	Foscan		650
BPD-MA	Verteporfin	Basal cell carcinoma	690
5-ALA	Levulan	Basal carcinoma, head and neck cancer, gynecological cancer	635
Mono-asparylchlorin e6 or Taloporfin Na, Npe6	Laserphyrin	Early endobronchial carcinoma. Preclinical studies	664
Disulfonate-Al phtalocyanine	Photosens	Head and neck. Preclinical studies	650–800
[5,10,15,20]-tetraks-m-hydroxil phenyl-chlorin	SQN 400 mTHPBC	Liver metastasis, preclinical studies.	740
Pd-bact-pheophorbide	Tookad	Prostate. Preclinical studies	763
2-[1-hexyloxyethyl]-2-divinyl pyropheophobide	HPPH Photochlor	Esophageal cancer. Preclinical studies	665
Sulfonated aluminium phtalocyanines	ALPcS_n_	Animal studies, cell lines	650–700
Hypericin		Cell lines	550–590
Indocyanine green	ICG	Cell lines	790
